# Cell-Phone-Based Platform for Biomedical Device Development and Education Applications

**DOI:** 10.1371/journal.pone.0017150

**Published:** 2011-03-02

**Authors:** Zachary J. Smith, Kaiqin Chu, Alyssa R. Espenson, Mehdi Rahimzadeh, Amy Gryshuk, Marco Molinaro, Denis M. Dwyre, Stephen Lane, Dennis Matthews, Sebastian Wachsmann-Hogiu

**Affiliations:** 1 Center for Biophotonics Science and Technology, University of California Davis, Sacramento, California, United States of America; 2 California Lutheran University, Thousand Oaks, California, United States of America; 3 California State University Sacramento, Sacramento, California, United States of America; 4 Department of Pathology and Laboratory Medicine, University of California Davis, Sacramento, California, United States of America; 5 Department of Neurological Surgery, University of California Davis, Sacramento, California, United States of America; University of California, United States of America

## Abstract

In this paper we report the development of two attachments to a commercial cell phone that transform the phone's integrated lens and image sensor into a 350× microscope and visible-light spectrometer. The microscope is capable of transmission and polarized microscopy modes and is shown to have 1.5 micron resolution and a usable field-of-view of 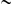
150×150 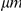 with no image processing, and approximately 350×350 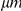 when post-processing is applied. The spectrometer has a 300 nm bandwidth with a limiting spectral resolution of close to 5 nm. We show applications of the devices to medically relevant problems. In the case of the microscope, we image both stained and unstained blood-smears showing the ability to acquire images of similar quality to commercial microscope platforms, thus allowing diagnosis of clinical pathologies. With the spectrometer we demonstrate acquisition of a white-light transmission spectrum through diffuse tissue as well as the acquisition of a fluorescence spectrum. We also envision the devices to have immediate relevance in the educational field.

## Introduction

With health care costs increasing throughout the world, there is a pressing need for reducing the cost and complexity of biomedical devices [1]. Additionally, with growing demand for high-quality health care in regions of the world where medical infrastructure is below levels found in developed countries, portable devices that can transmit relevant data to remote experts are likely to have a large impact on quantity and quality of care. To this end, several groups have focused on the development of low-cost and rapidly deployable technologies that address common diseases afflicting the third world and common tests performed in both hospital and field environments [1]–[6]. Cell phone cameras are certainly the most ubiquitous optical sensor in both the developed and developing worlds, and are attractive candidates for conversion to medical devices. Some work has already been directed towards this end, with several recent papers discussing the use of cell phones as diagnostic devices. Researchers at UCLA have constructed a modified lensless cell phone that enables holography-based digital microscopy [7], while researchers at UC Berkeley have constructed a complex objective attachment that also transforms a cell phone into a microscope [2]. Additionally, a patent was recently awarded for the use of a cell phone as a spectrometer [8]. However, there is still a need for more research directed towards utilizing cell-phone cameras to record images or spectra of biological samples.

The use of low-quality, low-cost components makes sense in the context of visual pathologic inspection. In this application, trained professionals manually examine samples to observe tissue- and cellular-level disorders, often with the aid of optical dyes. In fact, the fundamental basis of pathologic diagnosis has remained essentially unchanged for more than 100 years, following the standardization of staining procedures such as hematoxylin and eosin (H & E) for tissue sections and Wright-Giemsa staining for blood samples.

Additionally, the ability to cheaply and rapidly record diffuse reflectance spectra or fluorescence spectra also has the possibility to help with medical diagnosis. One example application is in the use of a spectrometer as a pulse oximeter, where the transmitted intensity through a finger is monitored and correlated through known absorption spectra to the concentration of oxy- and deoxy-hemoglobin. Additionally, a portable spectrometer might be used for the noninvasive detection of tumors, where it has been shown that tumors differ from surrounding healthy tissue by their increased autofluorescence and differing diffuse optical properties [9], [10].

In this paper we propose to take advantage of the rapid improvements in commercial CMOS sensors and microscopic optics driven by the cell-phone industry to develop two common biomedical devices, namely a microscope and spectrometer, that are available as simple and inexpensive add-ons to a commercial cell phone camera. While other researchers have demonstrated previously similar devices, our attachments to the phone are much smaller, simpler, and very low cost while still maintaining an acceptable level of performance. We demonstrate their relevance in laboratory measurements as well as discuss their applications within the field of science education.

## Results

### Cell Phone Microscope

#### Performance Tests

**Figure 1:**
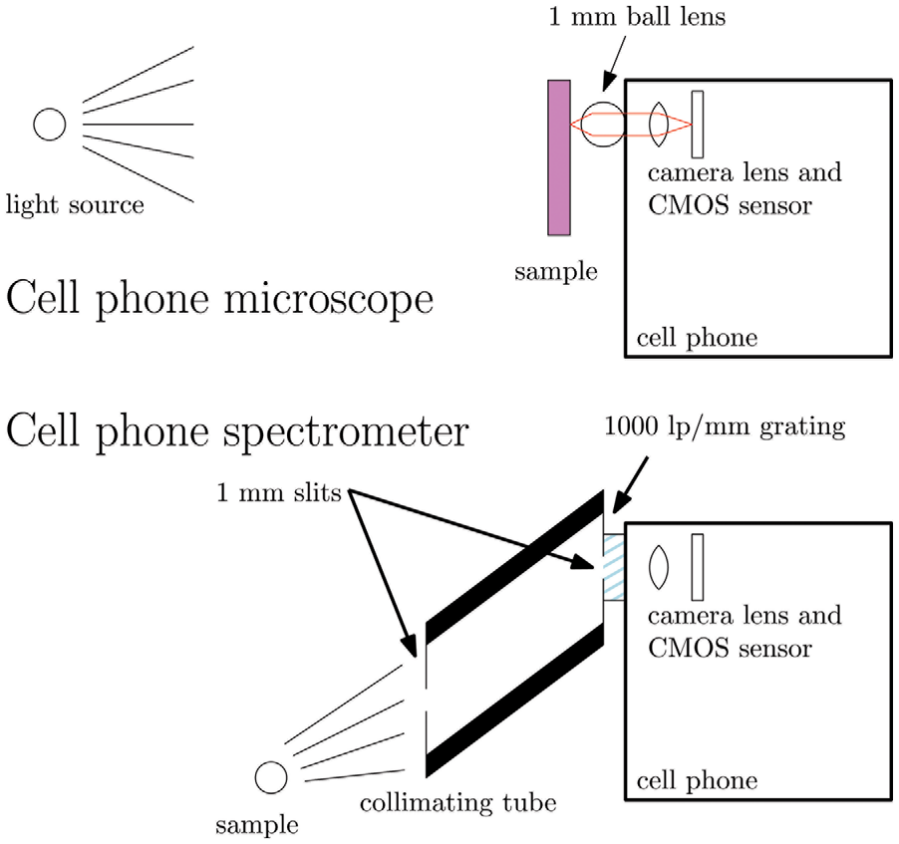
System diagrams. Top panel shows the cell phone microscope achieved by adding a ball lens to the cell phone camera system. Lower panel shows the cell phone spectrometer, constructed by adding a grating and collimating tube to the cell phone camera.

**Figure 2:**
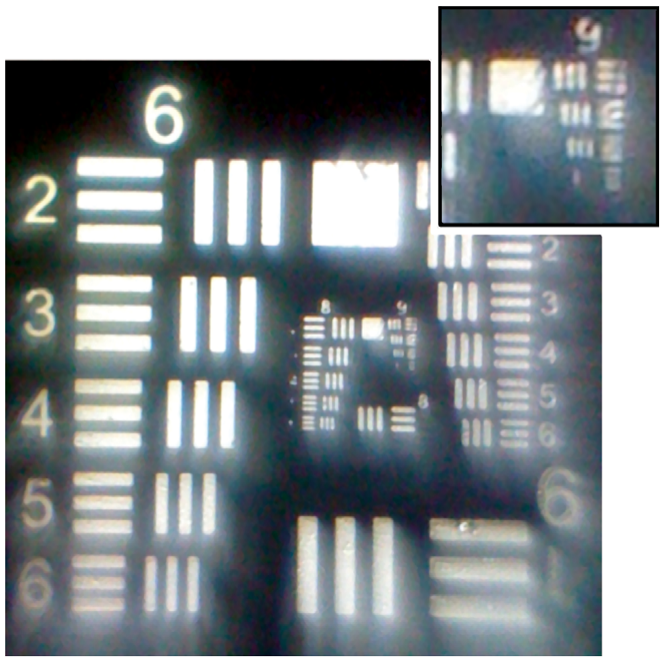
USAF resolution target. Image of resolution target taken with the iPhone 2G microscope, showing the ability to clearly resolve group 9 element 2, with slight distortions at the edge of the field.

A cell phone microscope was constructed as described in Materials and Methods and shown in Figure 1 To evaluate the performance of the cell phone microscope, and to characterize the extent of its optical aberrations, we took images of a 1951 USAF resolution target. The image taken by an iPhone 2G using a 1 mm ball lens is shown in Figure 2. Here we see that the system has nearly diffraction limited resolution, as the system can resolve Group 9, Element 2 of the target (corresponding to 575 lp/mm, or a resolution of approximately 1.5 microns).

**Figure 3:**
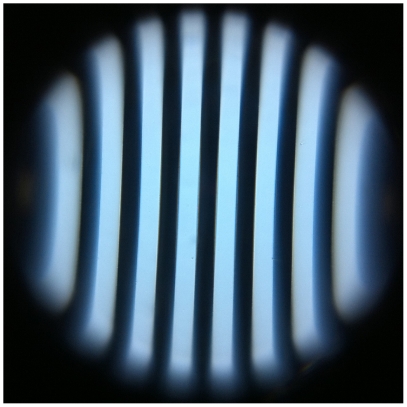
Cropped image of a 20 lp/mm Ronchi ruling using the iPhone 4 microscope. Pincushion distortion and defocus due to field curvature can clearly be seen at the edge of the field-of-view.

However, this resolution does not extend throughout the entire field-of-view. As can be seen in the test chart, the edges of the field have a significant defocus. This is due to our use of a simple ball lens as the magnifying element, which results in significant flat-field distortions. The lens's focal plane is described by a sphere, and the portions of the field that are in focus are those that intersect that sphere. Additionally, the system suffers from pincushion distortion, or field-dependent magnification. This can be seen clearly in Figure 3, where we image a Ronchi ruling of 20 lp/mm with the iPhone 4 microscope. Both of these aberrations can be characterized and corrected post-image acquisition through digital image processing.

**Figure 4:**
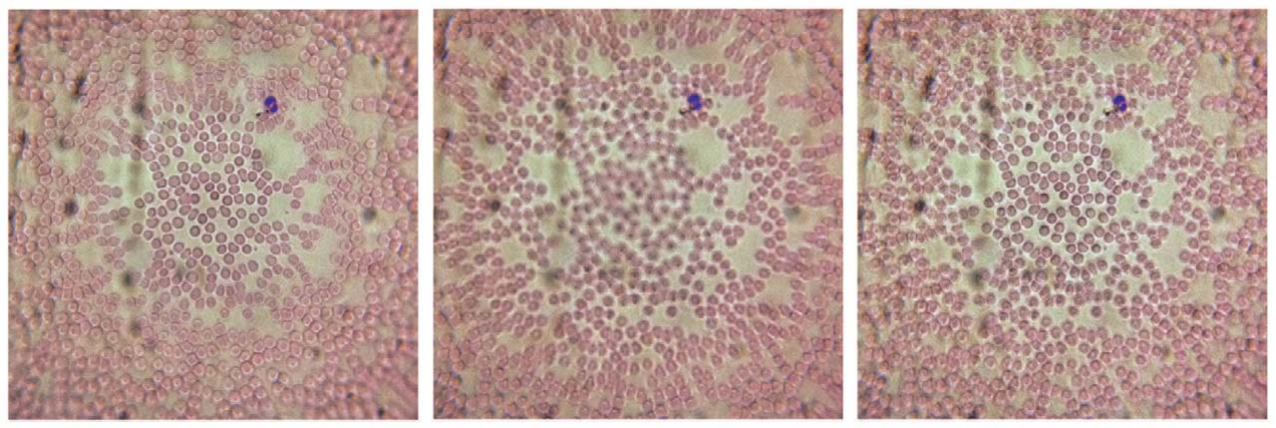
Demonstration of the multifocus fusion algorithm. Left panel: image of a Wright-Giemsa stained blood smear with the center of the field in focus. Center panel: image taken with the sample plane translated towards the phone by 2 micrometers. Right panel: Fused combination of previous two images, with fusion rule determined by the sum-modified Laplacian algorithm discussed in the text.

In this paper we use a sum-modified Laplacian based multi-focus fusion algorithm developed by Qu *et al.* [11]. Briefly, several images are recorded with varying focal planes and the contourlet transforms of these images are taken [12]. A focus metric known as the sum-modified Laplacian is calculated at each pixel in the contourlet domain [13]. A decision map in the transform domain is generated by determining which image has the maximum sum-modified Laplacian at each contourlet pixel. The image transforms are then fused based on this decision map. An inverse transform of this fused domain yields the final fused image. An example of the multifocus image algorithm applied to a Wright-Giemsa-stained peripheral blood smear is shown in Figure 4, where the images were recorded with the iPhone 4 microscope.

**Figure 5:**
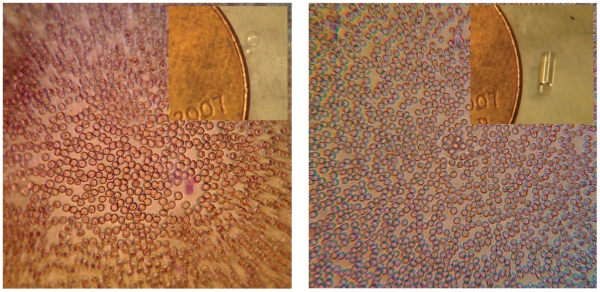
Micrographs of unstained peripheral blood smears taken with different lenses. Left panel: image taken with a 1 mm diameter ball lens, inset shows ball lens with penny as a reference. Right panel: image taken with a GRIN lens, inset shows size of GRIN lens with respect to a penny.

Additionally, we also examined the effect of different shapes and focal lengths of lenses. Some representative results of these studies applied to an unstained blood sample is shown in Figure 5. Here an image taken with a 1 mm ball lens is contrasted with that of an image of the same sample taken with a gradient index (GRIN) lens. We can see that although the GRIN lens provides an image with much less distortion and defocus aberrations, its magnification is lower and thus has a lower resolution than the best portions of the ball-lens image.

#### Imaging of Peripheral Blood Smears

**Figure 6:**
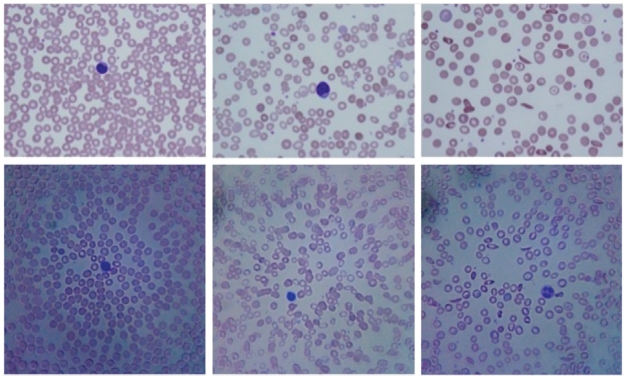
Micrographs of peripheral blood smears. Upper row: images from a traditional microscope. Bottom row: images from a cell phone microscope. Left, blood from a normal patient. Center, blood from a patient suffering from iron deficiency anemia. Right, blood from a patient suffering from sickle cell anemia.

Peripheral blood smears were taken from a patient with no blood-related illness, from a patient suffering from iron deficiency anemia, and a patient suffering from sickle cell anemia. The smears were prepared and stained with a modified Wright-Giemsa stain as discussed in the Materials and Methods section below. Images of the smears were recorded by a 20× microscope objective and by the iPhone 2G microscope equipped with a 1 mm ball lens. Those results are shown in Figure 6 with the upper row of images corresponding to the conventional microscope, and the lower row corresponding to the cell phone microscope. For simplicity, we have simply cropped the image to focus on the center region of the field-of-view, where the sample is in good focus. On the left we see a normal sample with the appropriate number of red blood cells and platelets. In the middle row we see the smear from the patient with iron deficiency anemia. Note the reduced number of red blood cells and the greater varying in cell size and shape compared to the normal patient. On the furthest right panel we see the patient suffering from sickle cell anemia. One can clearly see the characteristic banana-shaped sickled red blood cells indicative of the disease. Note in the right center of the cell phone image the clearly resolved nuclear structure of the polymorphonuclear leukocyte.

#### Automated Cell Counting

**Figure 7:**
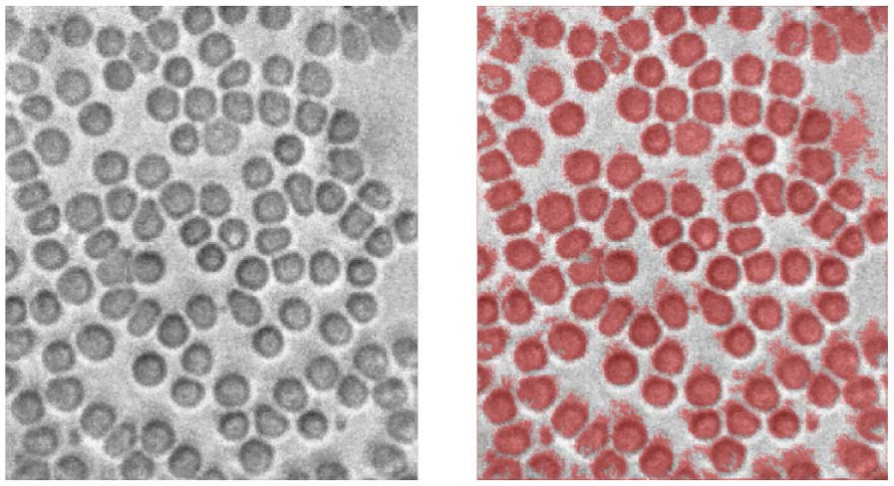
Automated counting and recognition of cells in a peripheral blood smear. Left panel, original image. Right panel, original image with objects identified as cells shaded in red. Counting done using CellC software.

One of our future goals is to develop a procedure to perform a partial or complete blood count. For the purposes of this paper, we show some preliminary data where we have cropped an image taken by the iPhone 4 microscope and explored automated image analysis methods to count cells. Results from an automated count utilizing the freely available CellC program developed by Selinummi [14] are given in Figure 7. The basis of this algorithm is an intelligent segmentation of the image into several clusters using marker-controlled watershed segmentation [15]. On the left hand panel of Figure 7 we see the original image submitted to the counting algorithm, while on the right we repeat the image overlayed with a mask defined by the segmentation algorithm. We see that all cells are correctly identified, along with some errors caused by a nonuniform background and the presence of small but optically dense platelets. Overall the algorithm reported a total cell count of 116, which agrees well with the hand-counted value of 113 (97% accuracy).

### Cell Phone Spectrometer

#### Performance Tests

**Figure 8:**
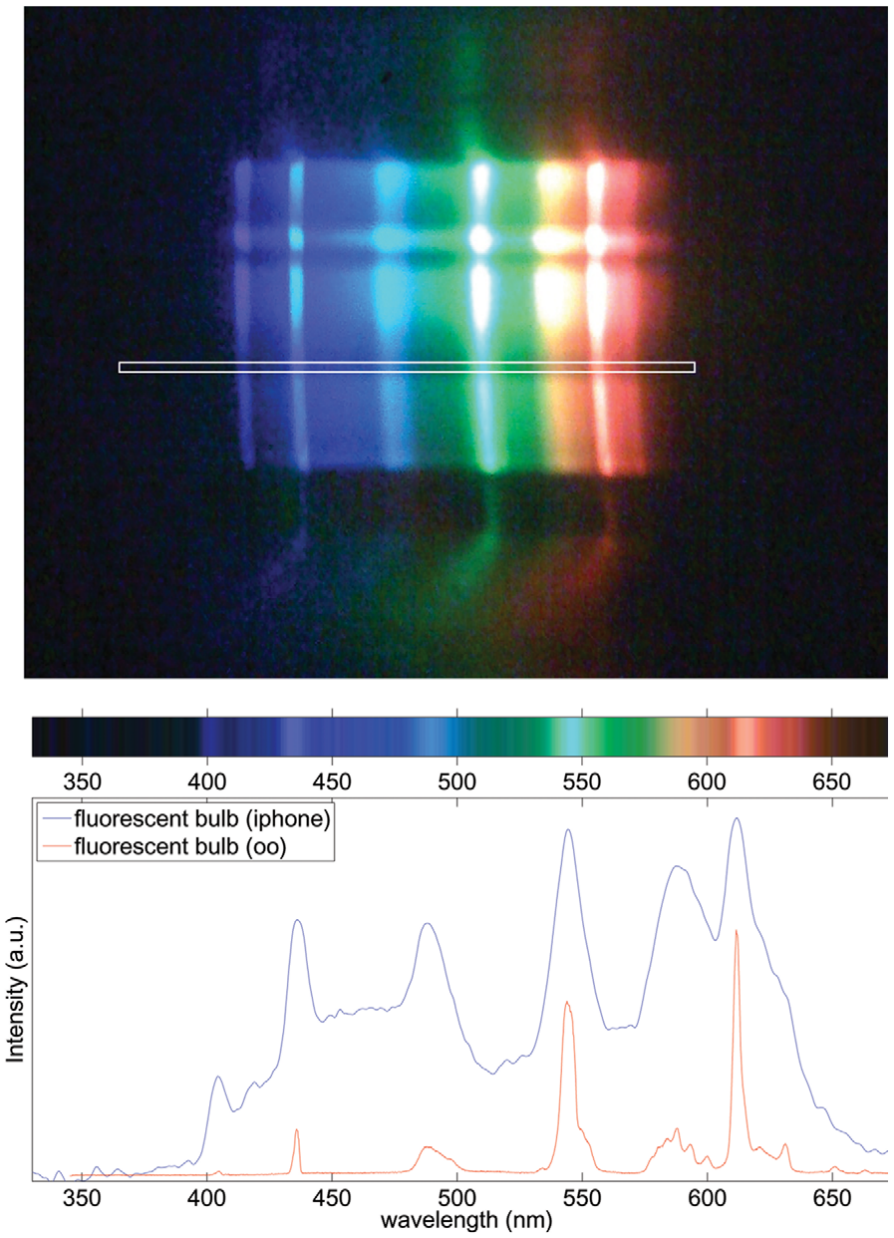
Image and spectrum of fluorescent bulb. Top panel, cropped image recorded by the cell phone spectrometer pointed at a standard fluorescent light fixture. White box indicates area used to determine spectrum in lower panel. Lower panel (top), an image of the spectrum corresponding to area in the white box in the top panel. Lower panel (bottom), a comparison of the spectra of the same fluorescent light fixture taken with both the cell phone spectrometer (blue) and Ocean Optics (oo) spectrometer (red).

The cell phone spectrometer was constructed as described in the Materials and Methods section, with a schematic diagram of the system shown in Figure 1. To characterize the performance of the cell phone spectrometer we simply pointed the cell phone at a fluorescent bulb featuring several narrow and intense peaks. The spectra were also taken using an Ocean Optics USB-4000. A cropped image taken by the spectrometer is shown in the top panel of Figure 8, where one can see the diffracted image of the slit. Taking just a single slice of the image (shown as a white box in Figure 8 ), we arrive at the spectrum shown in the bottom panel of Figure 8. Also shown in Figure 8 is the spectrum taken with the commercial Ocean Optics spectrometer, showing a good correspondence between the spectra. From this spectrum we can determine that the resolution of the system is approximately 10 nm. It is important to note that because the system is constructed by hand using household materials, the resolution and light-collection efficiency can be freely traded off. For example, by widening either the first or second slit, the overall light entering the system increases at the expense of spectral resolution. On the other hand, reducing the slit size increases the resolution up to approximately 5 nm (data not shown). 10 nm was chosen for this paper as it allowed us to rapidly collect data from several objects of interest at reasonable signal-to-noise ratios and reasonable spectral resolutions. Additionally, the system could be further improved by the addition of a lens within the tube to collimate light emerging from a single plane. This is done at the expense of the depth of field, which is essentially infinite in the current system. The infinite depth of field arises by similar means as the conceptually similar pinhole camera, in other words one may think of the spectrometer described here as the pinhole camera's spectral analog.

#### Transmission Spectrum through Tissue

**Figure 9:**
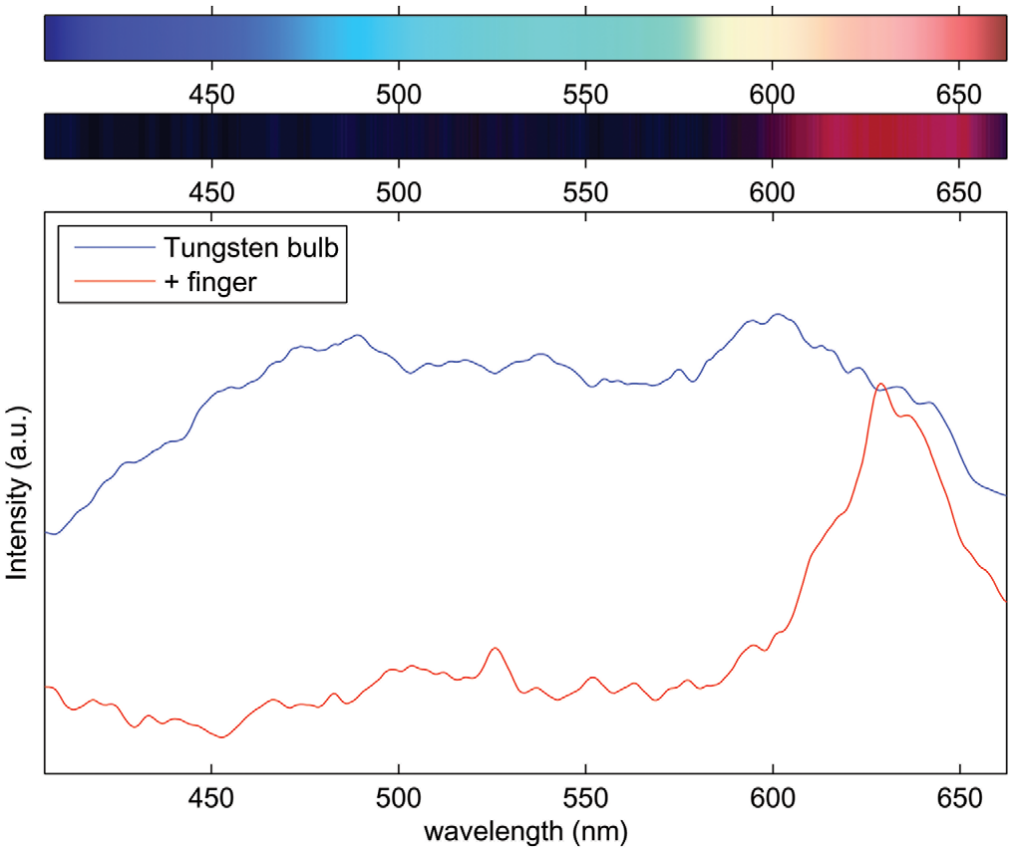
Transmission spectrum of a human finger. Upper panel, image of spectrum corresponding to a tungsten bulb. Middle panel, image of a spectrum corresponding to a tungsten bulb with a finger placed over the slit of the spectrometer. Lower panel, spectra of both the tungsten bulb and the transmission spectrum of the finger.

As one very simple biologically relevant experiment, we took a tungsten bulb and recorded its spectrum as a reference. Then, holding the distance between the camera and bulb fixed, a finger was inserted over the slit of the spectrometer, allowing one to record the transmission spectrum of approximately 1 cm of tissue. These results are shown in Figure 9. Note that in this case some autoscaling was done by the camera software outside the user's control. Thus we present these results in a qualitative sense only. However, obtaining user control over the camera's acquisition parameters is a direction we are currently pursuing. Despite this, the data point to the possibility of utilizing this spectrometer to make blood oxygenation measurements by recording differences in absorption in the finger at two wavelengths diagnostic of oxy- and deoxy-hemoglobin. Additionally, one could record the diffuse reflectance spectrum of tissue to characterize absorption and scattering using this device by coupling it with a simple fiber probe [10], [16]. As described in the previous references, in such an experiment, light propagated through a turbid tissue from an entrance point defined by an illumination fiber will tend to take an average path length through the tissue before exiting at an exit point defined by a collection fiber some distance removed from the illumination fiber. This average path length will depend on the scattering properties of tissue, and can be modeled by standard diffusion theory. By examining the absorption of the illumination light along that average path length, and knowing *a priori* the absorption spectra of several chromophores, one can quantify the concentration of those chromophores.

#### Fluorescence Spectroscopy

**Figure 10:**
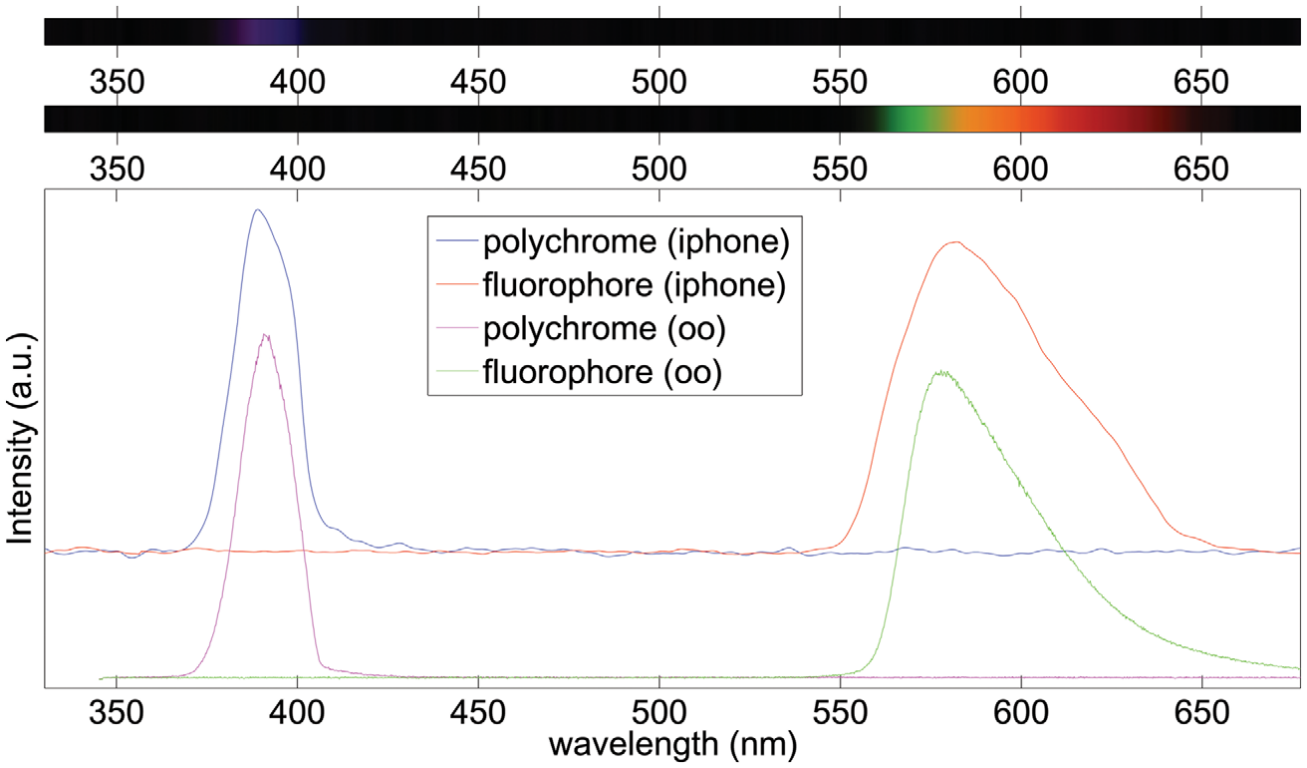
Fluorescence spectrum of rhodamine 6G. Upper panel, image of spectrum of Polychrome V light source. Middle panel, image of rhodamine 6G spectrum. Lower panel, a comparison of the excitation and emission spectra of the Polychrome V and rhodamine 6G taken with the cell phone spectrometer and validated with the commercial Ocean Optics spectrometer (oo).

Finally, we measured the fluorescence spectrum of rhodamine 6G excited with a Polychrome V light source tuned to 390 nm. The spectrum of both the light source and rhodamine fluorescence were measured by both the cell phone spectrometer and Ocean Optics spectrometer. Those results are shown in Figure 10. As can be clearly seen, the cell phone spectrometer results closely mirror those obtained with the commercial spectrometer. By measuring the fluorescence in a 90 degree geometry as described below in Materials and Methods, we avoid the use of costly optical filters. As is seen in Figure 10, the rhodamine fluorescence spectrum is free of any contamination by the Polychrome source. However, a filter to remove the excitation light could be easily inserted into the collection path, for example by placing it anywhere inside the PVC tube. Additionally, we stress that although the Polychrome source was convenient due to its presence in our lab, a high brightness LED or laser pointer could serve the same purpose, particularly since we did not collimate the output of the Polychrome V prior to measuring the rhodamine fluorescence. Such a cheap fluorescence system may be useful for diagnosis of lesions due to differences in their autofluorescence [17].

## Discussion

### Medical diagnostics

Although much important research has gone into developing very sophisticated diagnostic instruments, many important medical decisions are still based on expert opinions formed by trained professionals on the basis of data gathered via conventional devices such as microscopes, cell counters, and spectrophotometers. Replacing some of these costly and monolithic instruments with cheaper, portable devices that can achieve similar performance is an attractive option for reducing the cost and infrastructure burdens that quality health care places on society. Here we present two such devices integrated into a cell phone platform.

The first instrument, a cell phone-based microscope has been shown to have a resolution of 1.5 microns in the center of its field-of-view. Although the image quality rapidly degrades in a raw image due to the use of a single ball lens, the images can still be used to accurately diagnose a variety of blood diseases, as shown in Figure 6. We have explored other lens types, such as a cylindrical GRIN lens, to examine the effect of lens type and size on the image quality. Using a longer focal length ball lens, for example, results in a much larger field-of-view with fewer field-dependent aberrations, but at the expense of magnification and resolution (data not shown). In the case of a GRIN lens, the field distortions are much reduced at a slight expense of resolution (see Figure 5 ). However, a major impediment to the use of a GRIN lens is its extremely short working distance of just 50 microns. With this working distance the lens cannot image through a standard coverslip, severely limiting its utility in imaging blood smears and tissue sections. Additionally, we have shown that although the field-of-view has field dependent distortion and defocus when using the 1 mm ball lens discussed in this article, the aberrations are amenable to image processing. Specifically, we have shown that using a multifocus image fusion algorithm we have increased the usable field-of-view by a factor of approximately 2, as seen in Figure 4. We speculate that a robust program could be created that could take individual frames of a movie recorded by the phone and stitch those images together into a single image where the whole field-of-view is well focused. Through motion tracking and image fusion algorithms, the overall burden on the experimenter would be reduced and a high quality image could be obtained without needing to carefully mount or hold the sample with respect to the phone.

Furthermore, we have made an initial attempt at performing a red cell count of a blood sample imaged by the cell phone microscope. Although in this case we only report results of an algorithm locating and counting cells without regard to size or shape, the CellC algorithm or one similar could be easily used to report morphometric parameters that could enable an approximate complete blood count (CBC), discriminating cells into several blood cell classes.

The second instrument, a spectrometer consisting of a grating and collimating tube attached to the cell phone's camera, is shown to be capable of recording spectra with at least 5 nm spectral resolution, with resolution and light collection efficiency being freely traded off by the choice of the slit sizes. For the purposes of this paper we chose a slit size that gave us approximately 10 nm spectral resolution and allowed enough light throughput to easily record a fluorescence spectrum as well as a diffuse transmission spectrum. In all cases, the system compared well with the commercial Ocean Optics spectrometer in terms of spectral accuracy, with all peaks overlapping as expected after calibration.

Although our spectroscopic system may not currently have the throughput to measure more weakly fluorescing compounds, or obtain high quality diffuse reflectance or transmission spectra in the presence of low signal, these are actually not intrinsic limitations to the system. For example, with a diffuse reflectance system where source and detector are coupled to the tissue through optical fibers, a designated attachment could be designed that would obviate the need for the lossy collimation tube. Additionally, the collimation tube could be replaced by an inexpensive condenser assembly similar to those found in flashlights and car headlamps that would approximately collimate light. We are currently exploring these and other options to help improve the efficiency of the detection system.

We also note that the current state of the art of cell phone cameras are based on back-thinned CMOS sensors with 8-bit dynamic range. As scientific CMOS sensors become more ubiquitous, the cell phone will surely both drive and incorporate improvements in the detector industry and it may be that in the relatively near future commercial-grade camera sensors will approach the quality of detectors used in some less-demanding scientific applications today.

To conclude, we have presented above two devices built through adding simple and inexpensive attachments to a standard cell phone. We have demonstrated basic clinical utility of these devices through some initial experiments. We note that our choice of the iPhone as the camera for this work was driven primarily by the desire to have a camera placement that allowed easy lens attachment and sample viewing, as well as a touch screen interface to avoid motion due to button pressing. However, we do not believe the choice of phone to be crucial for this setup. We have replicated some of these experiments using other phones from different manufacturers with qualitatively similar results (not shown), indicating that the choice of phone and camera specifications are not the limiting factors in the performance of our system. These promising results will form the basis of future studies as we pursue more complex and rigorous evaluations of our devices as medical instruments.

### Education

#### Microscope

**Figure 11:**
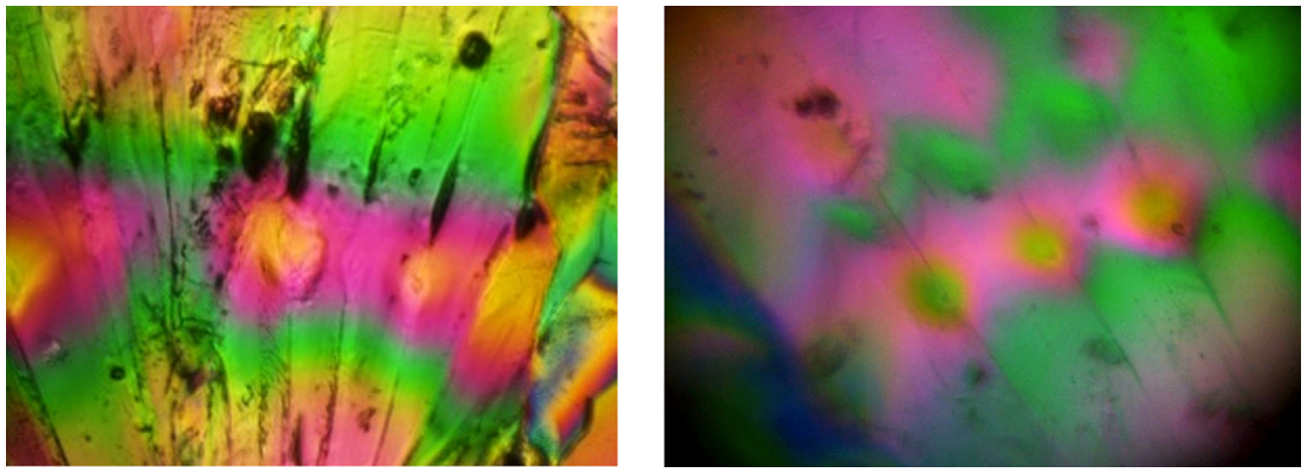
Polarized transmission images. Images of an sugar crystal taken through crossed polarizers. Left panel shows the image taken with a traditional microscope, right panel shows the image taken with the cell phone microscope.

**Figure 12:**
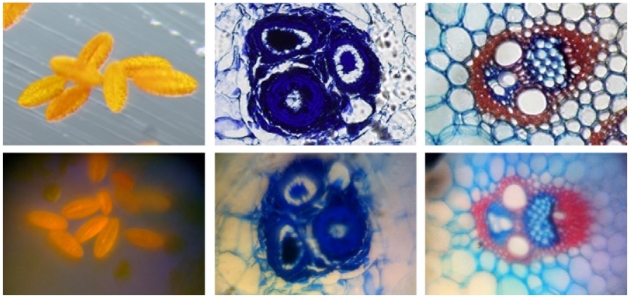
White light transmission images. Images of several commercially prepared microscope slides featuring stained samples. Top row, commercial microscope. Bottom row, cell phone microscope. Left column, pollen grains. Right two columns, plant stems.

The cell-phone microscope can work in multiple modes of operation, including polarized and transmission modes. The ability of the microscope to easily obtain simple but visually striking images points to the camera's usefulness as an educational tool. Here we present some example images taken with educational goals in mind. Polarized images of sugar crystals were taken by polarizing the incident illumination, and placing a second analyzer between the ball lens and iPhone 2G. The results are compared with a conventional polarized image of the same field-of-view using a 20× microscope and shown in Figure 11. The good correspondence between the two images shows that the microscope is behaving as expected within our restricted field-of-view. A similar filter approach could be used for obtaining fluorescence images for highly fluorescent samples. Figure 12 shows images taken of commercially prepared microscope slides featuring thin sections of biological samples stained with absorptive dyes. Different magnifications can be achieved by using ball lenses of different radii, and the ease with which lenses can be changed allows students to control the field-of-view.

The portability, low cost, versatility, and network connectivity of the cell-phone microscope point to potential uses in the primary, secondary and post-secondary educational environments. At the earlier ages, teachers, and students can take images of a variety of small objects from surfaces to insects that can be shared with the class. In the upper grades and college levels, the microscope can be used in the laboratory or field for obtaining close-up images of plants, thin mineral sections, rock surfaces and more that can be easily downloaded and shared as needed. As classroom microscopy equipment becomes more outdated and limited in number, the ability to perform a variety of microscopy experiments on a cell-phone becomes ever more relevant.

#### Spectrometer

The electromagnetic spectrum, especially the visible “color” portion, is a widely studied subject throughout the educational continuum appearing in science standards across the country. Spectrophotometry – the quantification of light energies generated by a lightsource, passed through a material, or reflected off a surface – appears in a variety of courses at the secondary and post-secondary levels. The cell-phone spectrometer provides a simple and inexpensive way to take a qualitative measurement of the energies of light in a given light “sample” that in current classrooms is either done with equipment costing in the thousands of dollars or is simply only talked about but never experienced. In sharing the approach with several teachers we have already witnessed the use of the cell-phone spectrometer to describe and discuss the properties of various light sources (LEDs, incandescent, fluorescent. Laser and other light sources), explain how the eye perceives color and can perceive different combinations of energies as constituting the same “color”, and measure simple fluorescence. We have tested the tool's capabilities in studying reflected light from dyes, plant materials, paints and more (data not shown) as well as for measuring the presence or absence of chlorophyll in leaves and other plant parts. Overall the tool performs at the level needed for educational use and is easy to make, use and share images collected. Additionally, analysis of images with simple and freely-available image analysis tools such as ImageJ provides educational opportunities in image processing and data analysis.

## Materials and Methods

### Ethics statement

The IRB (Institutional Review Board) Administration at University of California, Davis concluded that these studies are exempt from ethics review, as we used teaching samples from the Department of Pathology without personal identifiers associated with them, and samples obtained from one healthy volunteer in the lab, who is also an author on this paper. The volunteer from whom samples were collected provided both verbal and written consent for their blood to be used for this study.

### Pathology sample preparation

Sample hematopathology slides with known diseases were identified from the teaching collection of the Pathology Department at UC Davis Medical Center, and were prepared by standard procedures in the hematopathology laboratory at the University of California, Davis. The samples had no identifiers associated with them. Additionally, sample smear slides were prepared by collecting a single drop of blood from one healthy volunteer in the lab, who is also an author on this paper, using a finger stick. The drop of blood was placed on a glass slide and smeared using a second glass slide set on an edge and dragged across the first slide creating a wedge-shaped smear of blood. The slide is then allowed to air-dry and stained following the standard procedure for Wright-Giemsa staining using the equipment in the Pathology Department at UC Davis. First, the slide is fixed by dipping it into a methyl alcohol based fixative for 15 seconds. The slide is then immediately dipped into both a methylene blue nuclear stain for 15 seconds followed by a pink counterstain for 15 seconds.

### Cell phone cameras

Experiments utilized Apple-brand camera-enabled cell phones (iPhone 2G and iPhone 4G). The iPhone 2G employs a 2 megapixel CMOS sensor from Micron Technologies, Inc. with overall dimensions of 3.55×2.68 mm, and is comprised of 1600×1200 2.2 micron pixels. Each pixel is composed of a red, green, and blue-filtered sub-pixel. The camera has a single plastic biconvex lens with an effective focal length of 3.36 mm. The iPhone 4G utilizes a 5 megapixel CMOS sensor manufactured by LG Innotek. The camera has a physical size of 4.54×3.40 mm, with pixel dimensions of 2592×1944 composed of 1.75 micron pixels. The camera also features an autofocusing lens, also produced by LG Innotek.

### Cell phone microscope

#### Imaging lens

The construction of the iPhone microscope was as simple as adding a small ball lens mounted directly on top of the phone, as shown in the upper panel of Figure 1. A 1 mm diameter ball lens (Edmund Optics, Barrington, NJ) was mounted inside a small ring of black rubber to aid in light baffling. The rubber was then attached to the cell phone by means of double-sided tape. Performance targets were imaged by placing the sample in the plane of best focus.

#### Illumination considerations

**Figure 13:**
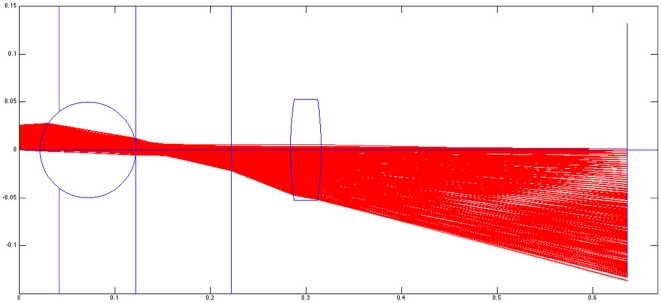
Raytracing simulation result. Trace of 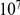 rays through a system composed of an 800 micron aperture, 1 mm ball lens, plastic biconvex lens, and CMOS sensor. Dimensions of plastic lens and sensor correspond to those in the iPhone 2G.

Illumination was achieved by means of white-light LED, which in some cases was covered by a piece of matte-finished adhesive tape acting as a low-grade diffuser. The LED was placed at a distance from the sample depending on the size and brightness of the source, attempting to achieve approximately collimated illumination across the field-of-view of the microscope. Simulations were performed to validate the construction of the microscope using in-house developed ray-tracing software running on the MATLAB platform (The MathWorks, Natick, MA). Simulation results demonstrated that using collimated illumination provides the system with an effectively infinite depth of focus at the expense of allowing all imperfections in the illumination beam path to imprint themselves sharply on the image. By contrast, increasing the divergence of the illumination decreases the depth of focus and allows more flexibility regarding the cleanliness and quality of the illumination path. However, as the illumination divergence increases, the portion of the field-of-view that is in focus also decreases. Figure 13 shows an example of a simulation using a 1 mm diameter spherical lens and a 0.8 mm diameter field aperture placed in contact with the iPhone 2G cell phone window. The illumination had a divergence of 5 degrees in this example. The lens displayed a 4.8× magnification and a field-of-view of approximately 0.55 mm diameter in the sample plane. The point of best focus was determined to be 0.722 mm in front of the lens, and the depth of field was approximately 3 microns. The numerical aperture of the system was calculated to be 0.44 leading to a diffraction limited resolution of .7 microns for 488 nm illumination. Because of the issues of artifacts contaminating the image when using collimated illumination, for the purposes of this paper we attempted to utilize an illumination source that roughly matched the 5 degree divergence simulated in Figure 13.

#### Polarized microscopy

Polarized images were acquired by placing a polarizer in front of the illumination source and an analyzer between the phone and the ball lens. Microscopic images were also taken for comparison on a commercial inverted microscope (BX-51, Olympus, Center Valley, PA) equipped with a 20× objective coupled to a commercial CCD camera (DP-71, Olympus, Center Valley, PA)

#### Sample holding

Sample holding was handled in two ways. In one scenario, where the ability to have fine control over focus adjustment was necessary ( Figure 4 ), the cell phone was held fixed while the sample was placed on a 3-axis translation stage to allow for very precise alignment. In other cases, the sample was held by hand and separated from the microscope by a rubber spacer that automatically placed the image in the proper plane such that the center of the field was in focus. Hematopathology samples were prepared as discussed above and placed in the plane of best focus.

### Cell phone spectrometer

The cell phone spectrometer is shown schematically in the lower panel of Figure 1 was constructed by first affixing a transmission grating (1000 lp/mm, Science Stuff, Inc., Austin, TX) over the window of an iPhone 2G's camera with standard tape. Two pieces of black electrical tape were then placed over the grating to form a slit of approximately 1 mm width. A 1/2-inch inner-diameter piece of PVC tubing was then lined with darkened foil (ThorLabs, Newton, NJ) to prevent inner reflections and a 7.75 cm long piece was cut at a 45 angle. This was then attached to the camera and another slit with a width of approximately 1 mm was formed at the distal end of the tube using the electrical tape. The tube, in combination with the two slits, acts to ensure that only approximately collimated light passes to the detector. For measurements of transmission through tissue, the spectrometer was pointed at a 60 W tungsten bulb and a finger was placed over the slit. For measurements of fluorescence, a bright, narrowband, tunable arc source (Polychrome V, TILL Photonics, Rochester, NY) tuned to 390 nm was directed at a cuvette containing 2 mL of a 1 mM solution of rhodamine 6G dissolved in water. The spectrometer was pointed at a face of the cuvette 90 from the direction of the 400 nm excitation propagation. A separate spectrum of the Polychrome source itself was taken by simply pointing the spectrometer at the source with no sample present. The resulting spectra were processed using MATLAB. Additionally, both the fluorescence and source spectra were acquired using a commercial spectrometer manufactured by Ocean Optics (USB-4000, Dunedin, FL) using fiber coupling.
